# Association of Leptin Gene DNA Methylation With Diagnosis and Treatment Outcome of Anorexia Nervosa

**DOI:** 10.3389/fpsyt.2019.00197

**Published:** 2019-04-11

**Authors:** Alexandra Neyazi, Vanessa Buchholz, Alexandra Burkert, Thomas Hillemacher, Martina de Zwaan, Wolfgang Herzog, Kirsten Jahn, Katrin Giel, Stephan Herpertz, Christian A. Buchholz, Andreas Dinkel, Markus Burgmer, Almut Zeeck, Stefan Bleich, Stephan Zipfel, Helge Frieling

**Affiliations:** ^1^Molecular Neuroscience Laboratory, Department of Psychiatry, Social Psychiatry, and Psychotherapy, Hannover Medical School (MHH), Hannover, Germany; ^2^Department of Psychiatry and Psychotherapy, Paracelsus Medizinische Privatuniversität Nürnberg, Nuremberg, Germany; ^3^Department of Psychosomatic Medicine and Psychotherapy, Hannover Medical School (MHH), Hannover, Germany; ^4^Department of Psychosomatic Medicine and Psychotherapy, University of Heidelberg, Heidelberg, Germany; ^5^Department of Psychosomatic Medicine and Psychotherapy, University Medical Hospital Tübingen, Tübingen, Germany; ^6^Department of Psychosomatic Medicine and Psychotherapy, LWL University Clinic Bochum, Bochum, Germany; ^7^Department of Psychosomatic Medicine and Psychotherapy, Klinikum rechts der Isar, Technical University of Munich, Munich, Germany; ^8^Department of Psychosomatics and Psychotherapy, University Hospital Münster, Münster, Germany; ^9^Department of Psychosomatic Medicine and Psychotherapy, Center of Mental Disorders, University Medical Center Freiburg, Freiburg, Germany

**Keywords:** leptin, leptin receptor, methylation, outcome, anorexia nervosa, epigenetic

## Abstract

Epigenetic alterations are increasingly implicated in the pathophysiology of anorexia nervosa (AN) but are as yet poorly understood. We investigated possible associations between the leptin gene (*LEP*) and the leptin receptor gene (*LEPR*) DNA promoter methylation and (1) a diagnosis of AN and (2) outcome after a 10 months psychotherapeutic outpatient treatment. 129 (*LEPR*: *n* = 135) patients with AN were investigated during the large scale psychotherapeutic Anorexia Nervosa Treatment Outpatient Study (ANTOP) trial, compared to 117 (*LEPR*: *n* = 119) age and height matched, normal-weight healthy controls. Blood samples were taken at baseline, the end of therapy (40 weeks) and the 12-months follow-up and compared to controls. Methylation was measured in whole blood via bisulfite sequencing. Within the promoter region 32 (*LEP*) and 39 CpG sites (*LEPR*) were analyzed. Two key findings were observed. First, *LEP* and *LEPR* methylation at baseline were lower in patients compared to controls (*LEP*: [%] AN: 30.94 ± 13.2 vs. controls: 34.53 ± 14.6); LEPR ([%] AN: 3.73 ± 5.4 vs. controls: 5.22 ± 8.3, mixed linear models: both *P* < 0.001). Second, lower DNA methylation of the *LEP* promoter, with a dynamic upregulation during treatment, was associated with a full recovery in AN patients (% change from baseline to follow-up in full recovery patients: +35.13% (SD: 47.56); mixed linear model: *P* < 0.0001). To test for potential predictive properties of mean *LEP* DNA methylation a *LEP* DNA methylation cut-off (31.25% DNA methylation) was calculated, which significantly discriminated full recovery vs. full syndrome AN patients. This cut-off was then tested in a group of previously unclassified patients (missing follow-up data of the Structured Interview for Anorexic and Bulimic disorders; *n* = 33). Patients below the cut-off (31.25% *LEP* DNA methylation) showed an increase in BMI over time, while those above the cut-off had a decrease in BMI (ANOVA at the 12-months follow-up: *P* = 0.0142). To our knowledge, this is the first study investigating epigenetic alterations in AN over time. Our findings indicate that *LEP* DNA methylation might be involved in the disease course of AN.

## Introduction

Anorexia nervosa (AN) is described as an eating disorder characterized by a persistent restriction of energy intake resulting in low weight, fear of gaining weight and a disturbed body image (1, 2). The disorder has a prevalence of 0.4% and one of the highest mortality rates in mental disorders (3). The costs it imposes to afflicted individuals, families, and society are high (4, 5).

Many studies suggest an influence of genetics on the etiology and pathophysiology of AN. Heritability rates range from approximately 56–74%, though, a distinct genetic pattern of AN could not yet be determined (6–8). The observed variance may be explained, at least in part, by the influence of environmental factors on gene expression and leads to a multicausal model of the development of the disorder.

Thus, epigenetic factors regulating gene expression and mediating the influence of environment on phenotype are of increasing interest in AN research (9).

In 2007 our group was the first to report alterations of DNA methylation in AN (10). Until now, all studies on DNA methylation in AN were cross-sectional studies comparing acutely ill or recovered women with healthy controls, thereby leaving open, which of the observed differences were due to starvation or might have been independent of body weight (11). Most studies were focusing on candidate genes, while five were investigating genome wide differences in DNA methylation levels with conflicting results [overview given in Table 1, (10–25)].

**Table 1:** Overview of DNA methylation studies in anorexia/bulimia nervosa.

**Gene**	**Meth**	**Subjects**	**Comment**	**Authors**
*SNCA*	**↑↔**	AN = 22 vs. HC = 30 BN = 24 vs. HC = 30		Frieling et al. (10)
*ANP*	**↔↑**	AN = 22 vs. HC = 30BN = 24 vs. HC = 30		Frieling et al. (12)
*BDNF*	**↔↑**	AN = 45 vs. HC = 45BN = 64 vs. HC = 32	Hypermethylation in BN associated with comorbid BPD and childhood abuse	Pjetri et al. (13)Thaler et al. (14)
*CNR1*	**↔**	AN = 20, BN = 23 vs. HC = 26		Frieling et al. (15)
*DAT*	**↑**	AN = 22, BN = 24 vs. HC = 30		Frieling et al. (16)
*DRD2*	**↑↔↔**	AN = 22 vs. HC = 30 AN = 45 vs. HC = 45 BN = 52 vs. HC = 19	Hypermethylation in BN associated with comorbid BPD	Frieling et al. (16) Pjetri et al. (13) Groleau et al. (17)
*DRD4*	**↔**	AN = 20, BN = 23 vs. HC = 26		Frieling et al. (16)
*HERP*	**↔**	AN = 22 vs. HC = 30 BN = 24 vs. H = 30		Frieling et al. (10)
*H19*	**↔**	AN = 10 vs. HC = 10	Buccal cells	Saffrey et al. (18)
*LEP*	**↔**	AN = 45 vs. HC = 45	AN partially weight-restored	Pjetri et al. (13)
*NR3C1*	**↔**	BN = 64 vs. HC = 32	PBMC's, hypermethylation with comorbid BPD, suicidality	Steiger et al. (19)
*OXTR*	**↑**	AN = 15 vs. HC = 36	Buccal cells	Kim et al. (20)
*POMC*	**↔**	AN = 22, AN weight-restored = 18 vs. HC = 24 AN = 40, AN weight-restored = 21 vs. HC = 54	PBMC's PBMC's, association with smoking	Ehrlich et al. (21)Ehrlich et al. (22)
*SERT*	**↔**	AN = 45 vs. HC = 45		Pjetri et al. (13)
Vasopressin	**↔**	AN = 22, BN = 24 vs. HC = 30		Frieling et al. (12)
**GENOME-WIDE STUDIES**				
Inverse assay with methylation (non-)sensitive restriction endonucleases	**↓↔**	AN = 22 vs. HC = 30 BN = 24 vs. HC = 30	Hypomethylation associated with elevated homocysteine	Frieling et al. (10)
LINE1 sequences	**↔**	AN = 10 vs. HC = 10	Buccal cells	Saffrey et al. (18)
Inverse assay with methylation (non-) sensitive restriction endonucleases	**↓**	AN = 32 vs. HC = 13	Adolescent patients, hypomethylation associated with leptin, and steroid hormone levels	Tremolizzo et al. (23)
450 K Illumina bead arrays	**↑**	AN-restrictive = 10, AN-binge/purge = 19 vs. HC = 15	PBMC's; no association with BMI; age of illness: gene pathways of brain development/ morphology duration of illness: gene pathways of anxiety, social functioning, physical complications of AN; OXT and 5-HT2A receptor	Booij et al. (24)
450 K Illumina bead arrays	**N/A**	AN = 47 vs. lean HC = 47 +100 HC (population-based), 5 AN discordant monozygotic twin pairs	Confirmation of TNXB hyper-methylation	Kesselmeier et al. (25)

*The literature search included combinations of the following terms: “methylation”/“epigenetic” and “eating disorder”/“anorexia”/“bulimia”. DNA Methylation was measured in whole blood if not otherwise stated*.

*AN, anorexia nervosa, BN, bulimia nervosa, HC, healthy controls, BPD, borderline personality disorder, PBMC, peripheral blood mononuclear cells, LINE1, Long Interspersed Nuclear Elements sequences. Genes: ANP, atrial natriuretic peptide; BDNF, brain derived neurotrophic factor; CNR1, cannabinoid receptor type 1; DAT, dopamine transporter; DRD2/4, dopamine receptor D2/4; HERP, homocysteine-induced endoplasmatic reticulum protein; H19, imprinted region near the insulin-like growth factor 2 gene; LEP, leptin; NR3C1, nuclear receptor subfamily 3, group C, member 1/glucocorticoid receptor; OXTR, oxytocin receptor; POMC, proopiomelanocortin; SERT, serotonin transporter; SNCA, synuclein alpha; TNXB, tenascin-XB; OXT, oxytocin; 5-HT2A receptor, 5-hydroxytryptamine receptor 2A/serotonin receptor 2A*.

The present study aims to analyze changes in DNA methylation of two candidate genes which have been previously implicated in the pathophysiology of AN, the *LEP* gene coding for leptin and the *LEPR* gene coding for the leptin receptor, in a longitudinal fashion.

Leptin, a hormone produced mainly in adipose tissue, acts in an anorectic manner, contributing to energy homeostasis via inhibition of Agouti Related Protein (AgRP) and Neuropeptide Y (NPY) neurons, while activating proopiomelanocortin (*POMC*) as well as cocaine and amphetamine regulated transcript (CART) neurons (26) in distinct areas of the brain, like the hypothalamic arcuate nucleus (ARC). The effects of leptin are mediated by the Leptin receptor, a class I cytokine receptor, via the JAK/STAT signaling pathway (27). Anorectic patients are suffering from hypoleptinemia with leptin serum levels being even lower than predicted from body fat mass (28, 29). Temporary hyperleptinemia upon refeeding in AN has been shown to be associated with renewed weight loss afterwards, shedding light on the importance of the underlying regulatory networks controlling leptin synthesis and secretion (30).

The expression of leptin is regulated by DNA methylation of the *LEP* gene promoter- which has been shown to be altered in obese patients or by perinatal undernutrition (31, 32).

The present study analyses *LEP* and *LEPR* DNA methylation in blood samples derived from a large scale psychotherapeutic trial recently conducted in Germany: To date the Anorexia Nervosa Treatment Outpatient (ANTOP) study (33) is the largest randomized-controlled multicenter outpatient trial in AN comparing cognitive behavioral therapy, focal psychodynamic therapy and optimized treatment as usual. For the present analysis, DNA methylation of AN patients of the ANTOP study was studied at baseline, at the end of therapy, at the 12-months follow-up and compared to age and body height matched healthy control women. We hypothesized that AN patients will have a higher *LEP* DNA methylation (reflecting transcriptional silencing) and a lower *LEPR DNA* methylation (reflecting up-regulation of the receptor) when compared with controls and that these differences will diminish during therapy and be sustained in case of remission.

## Material and Methods

### Participants

We measured the promoter DNA methylation of the leptin gene in 129 female adult patients suffering from AN and 117 age and height matched healthy controls, as well as the DNA methylation of the promoter of the leptin receptor of 135 patients and 119 healthy controls. The different numbers of patients and controls between *LEPR* and *LEP* were caused by exclusion of 6 *LEP* patients and 2 *LEP* controls due to failure of DNA methylation measurement (PCR, sequencing). Patients participated in the ANTOP study conducted by the Eating Disorders Diagnostic and Treatment Network EDNET group [for study protocol and results, see (34) and (33)]. Healthy controls were recruited in Hannover via advertisements and did not receive financial or any other compensation for their participation.

The study adhered to the Declaration of Helsinki (1964) and its later amendments. Independent research ethics committees at every participating center approved the ANTOP study. Approval for the control study was obtained at the Ethics Committee of the University of Hannover (Permit Number 6427). Written informed consent was obtained from all patients and controls after the procedures had been fully explained to them and prior to their inclusion in the study.

Controls received a brief medical examination and answered patient's questionnaires. They were excluded from the control group if one or more of the following circumstances occurred: BMI ≤19 or ≥25, eating disorder, other psychiatric disorders, pregnancy, current somatic disorder. For comparison between patients and controls, as well as for the assessment of exclusion criteria patients and controls completed the Eating Disorders Inventory: EDI-2 (35) and the Patient Health Questionnaire: PHQ (36). Patients were additionally assessed by the Structured Interview for Anorexic and Bulimic disorders: SIAB-Ex, which was conducted by trained research assistants, while controls only answered the SIAB questionnaire (37).

As described in detail in the original study report, severity of AN and outcome were measured by the Psychiatric Status Rating (PSR) Scale based on the patient's SIAB-EX interview. PSR scores range from 1 (no symptoms of AN) to 6 (severe symptoms of AN that require admission). A score of 5 indicates that all DSM-IV criteria for AN are fulfilled. A global outcome score (end of treatment after 40 weeks and 12-months follow-up visit) was established according to the original ANTOP Trial based on the following combinations of PSR scores and BMI: full recovery was defined as a PSR score of 1 or 2 and BMI >18.5 kg/m^2^; full syndrome anorexia nervosa as PSR score of 5 or 6 and BMI of 17.5 kg/m^2^ or lower; and partial syndrome AN included all other cases (33).

### DNA Preparation and Bisulfite Sequencing

For DNA isolation, blood samples were drawn from patients at baseline, at the end of treatment (after 40 weeks), and at the 12-months follow-up visit. Blood of healthy controls was drawn at baseline.

The following extraction of DNA was done with the QIAamp DNA Blood Mini Kit by Qiagen (Hilden, Germany), using 200 μl of Ethylenediaminetetraacetic acid blood samples and 15 min of incubation at 56°C. Tubes were left open after step 10 (removal of possible washing buffer carryover) for 5 min at ambient temperature. After that, DNA was eluted twice: first in 100 μl, followed by 50 μl elution buffer. Columns were incubated with elution buffer 5 min prior to centrifugation.

For discrimination of methylated and unmethylated cytosines, isolated DNA underwent subsequent bisulfite reaction via the Qiagen Epitect® Bisulfite 96 Kit (Qiagen, Hilden, Germany). Upon this treatment unmethylated cytosines deaminate into uracils whereas methylated cytosines remain unaffected.

Bisulfite treated DNA was used to perform nested PCR specific for the *LEP* and *LEPR* gene to obtain products of 702 and 460 bp, respectively (see Supplemental Figures S1A,**B** for sequences and CpG positions). Supplemental Tables S1A,B show PCR conditions and primers in detail.

**Figure 1:**
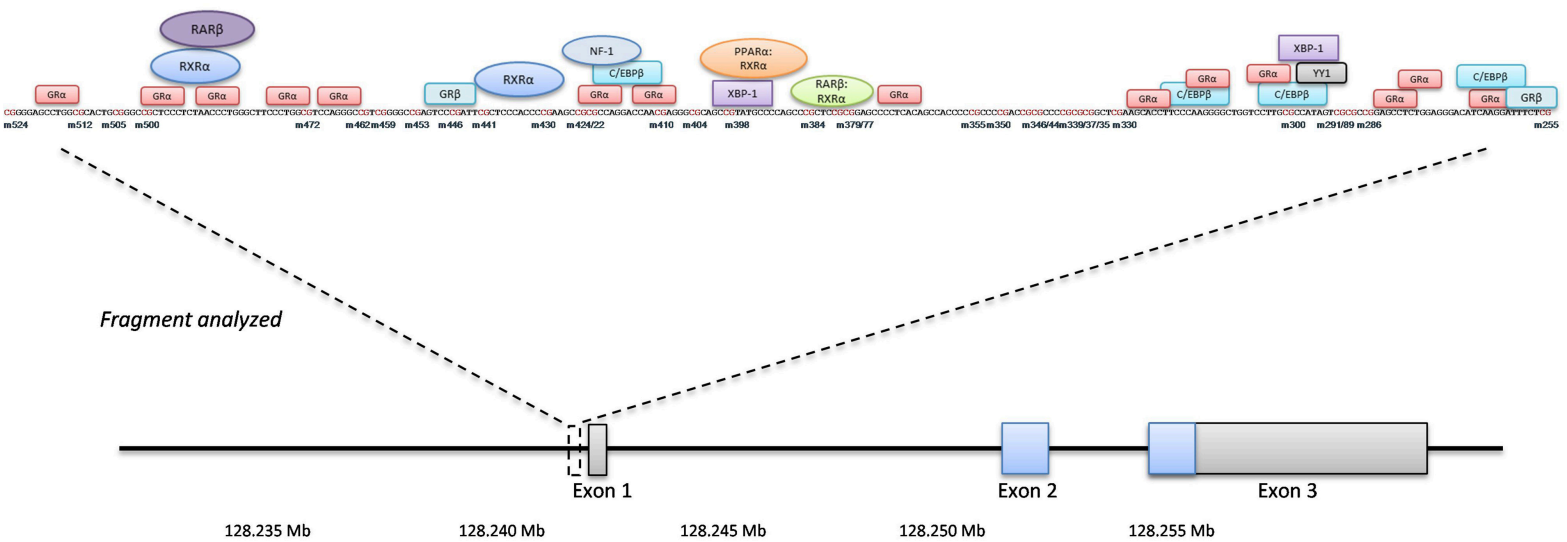
***LEP***gene overview. The position of the analyzed fragment within the *LEP* gene including the single investigated CpG sites (numbering is minus to exon I). Putative transcription factors and their binding sites are shown in boxes and circles.

Figure 1 gives an overview of the studied fragment with its position within the *LEP* gene, studied CpG's and the putative transcription factor binding sites predicted by PROMO applying a 10% maximum dissimilarity matrix (http://alggen.lsi.upc.es/cgi-bin/promo_v3/promo/promoinit.cgi?dirDB=TF_8.3). Amplification products underwent automated clean-up on a Biomek® NXP by Beckman Coulter using Agencourt® AmPure® XP magnetic beads (Beckman Coulter, A63881). Detection of products was done via agarose gel electrophoresis, followed by sequencing-PCR according to Sanger, using the Big Dye® Terminator v3.1 Cycle Sequencing Kit as described in the manufacturer's protocol and a maximum amount of 30 ng DNA (Applied Biosystems). Products were again cleaned up by the Biomek® NXP, this time using Agencourt® CleanSeq® XP magnetic beads (Beckman Coulter, A29154), and subsequently used for sequencing (Genetic Analyzer 3500xL, Applied Biosystems). All PCRs were performed on a C1000™ ThermoCycler (BioRad), analysis of sequencing results and determination of the methylation rate at every single CpG site was done by the specialized Epigenetic Sequencing MEthylation Analysis software v3.2.1 (ESME, Epigenomics AG). In our hands, this approach results in variability (precision measured with repeated measurements of the same samples for *LEP* and *LEPR*) of around 2.5% and an accuracy between 1 and 4% (comparing measurements against artificially methylated DNA).

### Statistics

Bisulfite sequencing yielded DNA methylation values of 41 single CpG sites in the *LEP* gene and 45 single CpG sites in the *LEPR* gene. We performed initial quality checks to exclude potentially unreliable measurements: (a) All obtained sequences were screened for sequencing quality using (I) manual inspection of the traces in Genious (Biomatters ApS, Aarhus, DK) and using ABI sequence scanner (Applied Biosystems). Samples with a QV-value <20 were measured again. For the final analysis, all sequences were above the QV threshold of 20. (b) Individual CpG sites with more than 5% missing values were excluded from the analysis, leaving 32 CpG sites (*LEP*) and 39 CpG sites (*LEPR*). CpGs that had to be excluded were located in the read-in and read-out parts of the fragments. (c) All study participants had <10% missing values for each gene. (d) CpG sites with an inter-individual variation below 0.01 were excluded from the analysis see also (38). All 32 CpG sites in the *LEP* gene fulfilled this inclusion criterion, while for *LEPR* 6 CpG sites were excluded leaving 33 CpG sites in the final analysis of this gene. Correlation matrices between CpG sites of *LEP* and *LEPR* are provided in the supplements.

Mixed linear modeling (REML) was used to test for association between DNA methylation and different predictors. To do this, first empty models were calculated (with only CpG-position (model I) or CpG-position and timepoint (model II) as repeated measures and subject-ID as random effect) and different covariance structures were compared using Akaike's Information criteria (AIC). In all four models tested, a scaled identity covariance structure showed the best fit (data provided in the Supplemental Statistical Tables S2).

Mixed linear modeling was performed computing baseline DNA methylation as dependent and (model I) group (patients vs. controls), age and BMI as fixed effects. Subject-ID was treated as random effect. CpG position was entered as repeated measurement using scaled identity covariance structure.

To test the effect of outcome on DNA methylation, we used mixed linear modeling (II) in patients only, computing methylation as dependent variable and timepoint, global outcome (at 12-months follow-up), and timepoint x global outcome interactions and age and BMI as fixed effects. CpG and timepoint were computed as repeated measurements using scaled identity covariance structure. Estimated marginal means were calculated for group (model I) or timepoint x global outcome interaction (model II) and compared by Sidak's *post-hoc* test. Parameter estimates were calculated for all factors and manually inspected. Model fits were compared using the−2loglikelihood ratio.

Significant global outcome x timepoint interactions were further analyzed using mean promoter DNA methylation as dependent variable at the different timepoints separately in a one-way analysis of variance (ANOVA) followed by Sidak's *post-hoc* test. Predictive properties of baseline DNA methylation of *LEP* were tested using logistic regression and subsequently receiver-operator-characteristics (ROC) curve, the area under the curve (AUC) was calculated for the contrast between full recovery AN (full response) and full syndrome AN (non-response) based on the global outcome. Youden's index (YI) was calculated as the mean *LEP DNA* methylation with the highest combined sensitivity and specificity. This cut-off was then tested for differences in the BMI trajectories of 33 study participants with missing PSR ratings, that were not included in the previous analyses (except model I) using two-way ANOVA with time (as repeated measure) and *LEP* DNA methylation above or below YI as factors and BMI as dependent variable, again followed by Sidak's *post-hoc* test. For all analyses if not otherwise stated, a *P*-value below 0.05 was considered significant. Given that the lower precision margin of our bisulfite sequencing is around 2.5%, we decided to regard only statistically significant differences between groups above 2.5% as clinically relevant. All analyses were performed using IBM SPSS™ 24 for Windows and GraphPad Prism 6.0.

## Results

### Patient Characteristics

Demographic data of patients and age-matched healthy controls are shown in Table 2. At baseline, all patients had a PSR score of 4 (subsyndromal AN) or 5 (full syndromal AN), data on global outcome at the 12-months follow-up were available in 93 patients (a comparison of baseline characteristics of these 93 patients compared to the 36 patients without PSR ratings at the follow-up is provided in Supplemental Table S3).

**Table 2:** Characteristics of patients and healthy controls.

**Characteristics, mean (SD)**	**Patients (*n* = 129)**	**Global outcome of patients at the 12-months follow-up (*****n*** **=** **93)**			**Controls (*n* = 117)**
		**Full syndrome AN (*n* = 23)**	**Partial syndrome AN (*n* = 48)**	**Full recovery (*n* = 22)**	
Age, years	27.31 ± 7.6	30.26 ± 9.31	26.84 ± 6.84	24.92 ± 5.97	29.47 ± 9.3
BMI, T0	16.8 ± 0.94	16.26 ± 0.94	17.08 ± 0.85	17.03 ± 0.82	21.7 ± 1.8
BMI, T2 (*n* = 112)	17.6 ± 1.72	16.23 ± 0.98	17.86 ± 1.46	18.89 ± 1.76	
BMI, T3 (*n* = 101)	18.19 ± 2.03	15.69 ± 0.94	18.52 ± 1.3	20.3 ± 1.49	
Subtype, *n* (%)					
Binge-purge AN	63 (48.8)	12 (52.2)	28 (58.3)	6 (33.3)	
Restrictive AN	66 (51.2)	11 (47.8)	20 (41.7)	12 (66.7)	

*Characteristics' of patients and healthy controls are shown for those with available LEP promoter DNA methylation measurements. T0 indicates the baseline measurement, T2 the end of treatment after 40 weeks, T3 the 12-months follow-up visit. Full syndrome AN patients had a BMI ≤ 17.5 and a PSR of 5 or 6, full recovery patients a BMI > 17.5 and a PSR of 1 or 2*.

### Differences in *LEP* and *LEPR* DNA Methylation Between Acute AN and Healthy Control Women

**Figure 2:**
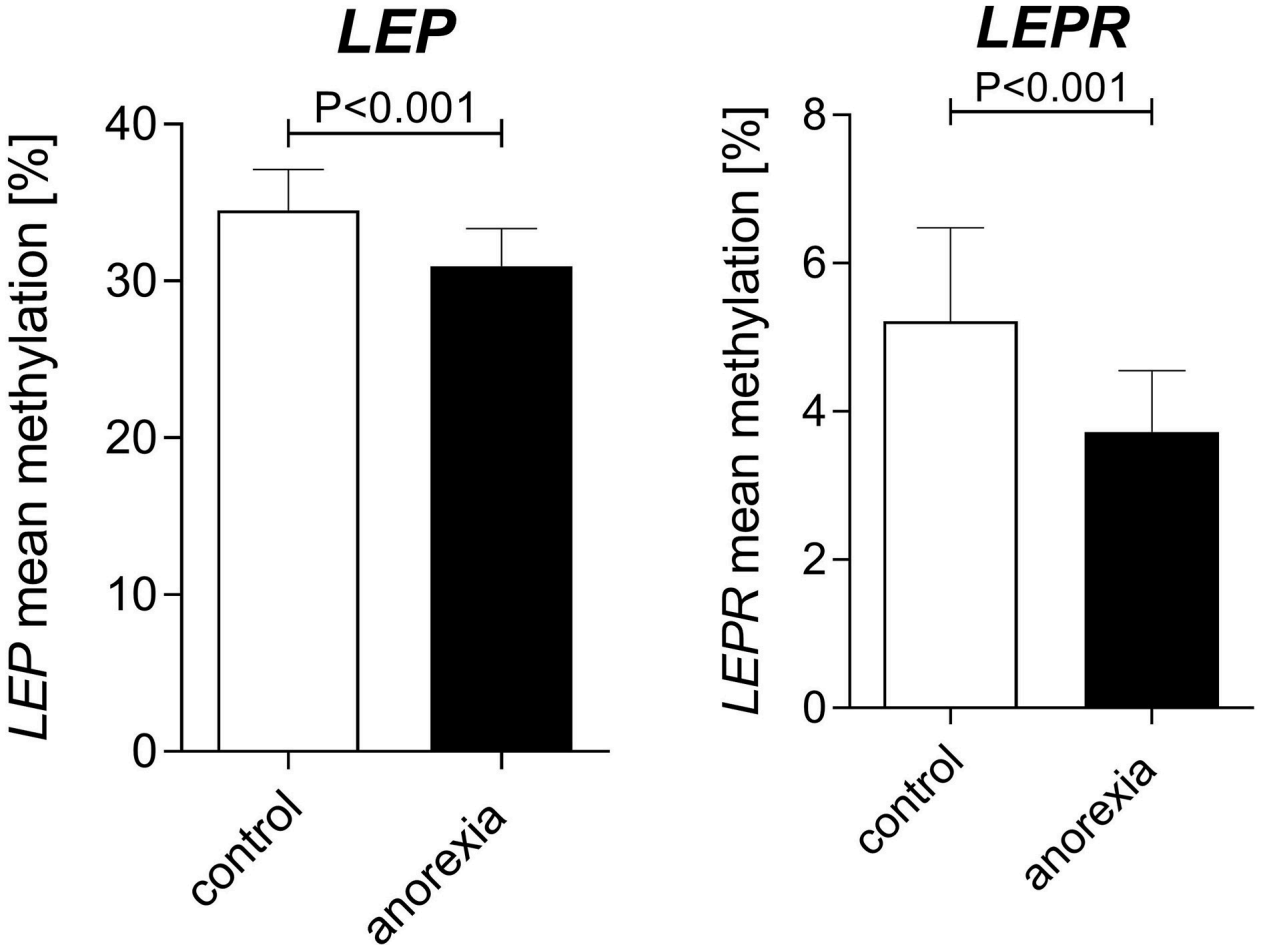
Baseline differences in *LEP* and *LEPR* DNA methylation between adult women with AN and healthy controls. At baseline AN patients had a lower DNA methylation of the *LEP* and *LEPR* promoter compared to age-matched healthy women. Raw mean values are shown; error bars show the standard deviation. *P*-values are derived from mixed linear modeling. Further details are summarized in the results section.

Adult women with acute AN at baseline had lower levels of both *LEP* ([%] AN: 30.94 ± 13.2 vs. controls: 34.53 ± 14.6) and *LEPR* ([%] AN: 3.73 ± 5.4 vs. controls: 5.22 ± 8.3) promoter DNA methylation when compared with healthy control women (Figure 2, mixed linear model analysis I: both *P* < 0.001). In detail, we found significant associations of group and age with *LEP* DNA methylation, while BMI showed no association in the mixed linear model analysis. The Supplemental Figure 3 shows the differences in methylation at the single CpG sites for AN and the healthy control group at baseline. Regarding *LEPR* DNA methylation, we found significant associations of group and BMI, while age had no association with *LEPR* methylation. Details of the mixed linear model I are provided in Table 3.

**Table 3:** Details of the mixed linear model comparing patients and controls at baseline (model I).

**Gene**	**Predictor**	**Parameter estimate**	**Standard error**	**df**	***T*-value**	***P*-value**
*LEP*	Intercept	30.75	2.84	6903	10.84	< 0.001
	Group	3.80	0.95	6903	4.00	< 0.001
	BMI	−0.11	0.17	6903	−0.63	0.532
	Age	0.08	0.03	6903	2.63	0.008
	AIC: 2780.59; No of parameters: 5; No of observations: 216; Estimated covariance parameter (repeated measurement): 3.90 (s.e.: 0.06)					
*LEPR*	Intercept	10.91	2.00	7392	5.46	< 0.001
	Group	2.56	0.52	7392	4.94	< 0.001
	BMI	−0.19	0.09	7392	−2.02	0.044
	Age	−0.01	0.01	7392	−0.62	0.532
	AIC: 11 275.50; No of parameters: 5; No of observations: 216; Estimated covariance parameter (repeated measurement):1.27 (s.e.: 0.03)					

*Dependent variable: methylation [%]*.

### Treatment Outcome and DNA Methylation Trajectories Over Time

**Figure 3:**
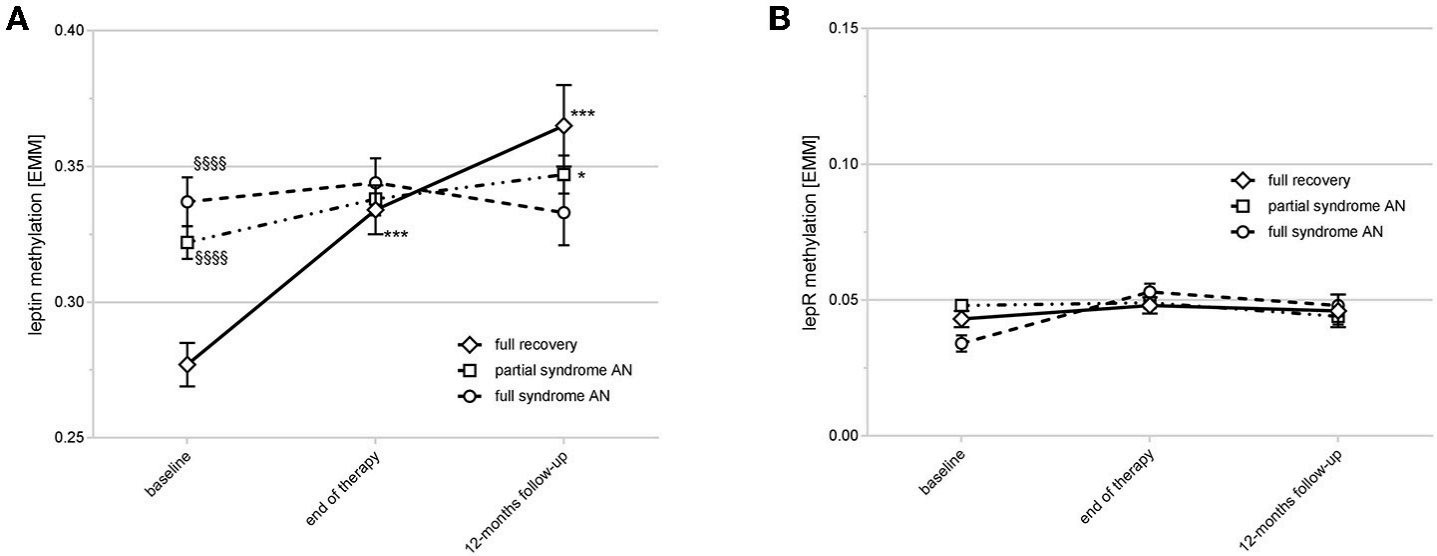
Time course of *LEP* and *LEPR* DNA methylation and outcome at the 12-months follow-up. **(A)** shows the time course of *LEP* DNA methylation in different response types. Full recovery patients had a lower DNA methylation of *LEP* compared to partial/full syndrome AN patients at baseline (^§§§§^*P* < 0.0001) with an increase at end of therapy and the 12-months follow-up (***P < 0.0001). Partial syndrome AN patients had a marginal increase of DNA methylation at the follow-up (*P < 0.05). *P*-values are derived from Sidak's *post-hoc* test after mixed linear modeling. Estimated marginal means [EMM] are shown; error bars show the standard deviation. **(B)** shows the *LEPR* DNA methylation over time with no clinically relevant differences between the timepoints or outcome types. Estimated marginal means [EMM] are shown; error bars show the standard deviation.

PSR ratings and BMI for all three time points were only present in 93 of the AN patients (“discovery sample”). As shown in Figure 3A, patients fully recovering during therapy and showing the best outcome at the 12-months follow-up had the most dynamic modulation of *LEP* DNA methylation. They started with significantly lower levels at baseline compared to patients without full recovery (significance levels of Sidak-corrected *post-hoc* tests are provided in Figure 3A). During treatment and the follow-up period this group of patients had a significant increase (% change from baseline to follow-up: +35.13% (SD: 47.56); mixed linear model II: *P* < 0.0001) in *LEP* DNA methylation resulting in significantly higher methylation levels at the follow-up visit when compared to the partial and full syndrome AN patients (Figure 3A). This increase in DNA methylation was already significant at the end of treatment (% change from baseline to end of treatment: +29.15% (SD: 55.7); mixed linear model II: *P* < 0.0001) and even more pronounced at the 12-months follow-up (Figure 3A). Partial syndrome AN patients also showed a significant, hence compared to the full recovery AN group, only marginal increase from baseline to follow-up (% change from baseline to follow-up: +16.61% (SD: 56.37); mixed linear model II: *P* < 0.05; Figure 3A), while DNA methylation levels of the full syndrome AN group remained stable (% change from baseline to follow-up: −2.05% (SD: 27.20); P: n.s.). In comparison, no clinically relevant changes in DNA methylation of the *LEPR* gene were found (Figure 3B). The Supplemental Tables S4, S5 provide the statistical information about the mixed linear model II for *LEP* and *LEPR*. The Supplemental Figures S4A–C show the differences in methylation at the single CpG sites for AN groups at baseline (Supplemental Figure S4A), end of therapy (Supplemental Figure S4B) and follow-up (Supplemental Figure S4C).

To test for potential predictive properties of mean *LEP* DNA methylation, we used logistic regression analysis entertaining the BACKWARD method, computing outcome (full recovery vs. full syndrome at follow-up) as dependent variable and age, baseline BMI, subtype (bulimic vs. restrictive type) and mean *LEP* methylation at baseline as predictors. The final model including LEP methylation, BMI and subtype accurately predicted the outcome in 86.5% of all cases, while the empty model classified all patients into the full recovery group (accuracy: 51.4%). A summary of the logistic regression results is provided in Table 4.

**Table 4:** Logistic regression on outcome at follow-up (full recovery vs. full syndrome AN).

	**R^2^_**(*Nagelkerke*)**_**	**Accuracy [%]**	**Predictors**	**B**	**Wald**	**df**	***P*-value**
**STEP 2**	0.454	86.5	Intercept	−14.54	2.24	1	0.135
			LEP methylation	−17.46	4.87	1	0.027
			BMI	1.24	4.12	1	0.042
			subtype	−1.80	3.24	1	0.072

To further analyse this finding, we performed a ROC-curve analysis, which revealed a moderate but significant ability to discriminate between later full recovery AN vs. full syndrome AN patients (AUC: 0.737 (s.e.:0.086); *P* = 0.014). Cumulative distribution analysis revealed that the cut-off value with the best combined sensitivity and specificity (Youden's Index, cut-off A) was at a mean DNA methylation of 31.25% (see Supplemental Figures S2A,B). A second cut-off value (cut-off B) was selected with the highest sensitivity at specificity of 0.8, which we found to be at a DNA methylation level of 27.77%. In all subsequent analyses, cut-off A outperformed cut-off B, therefore only results for cut-off A are reported.

Next, we aimed to test our calculated cut-off in a group of patients with missing PSR ratings to validate our finding (“replication sample”).

**Figure 4:**
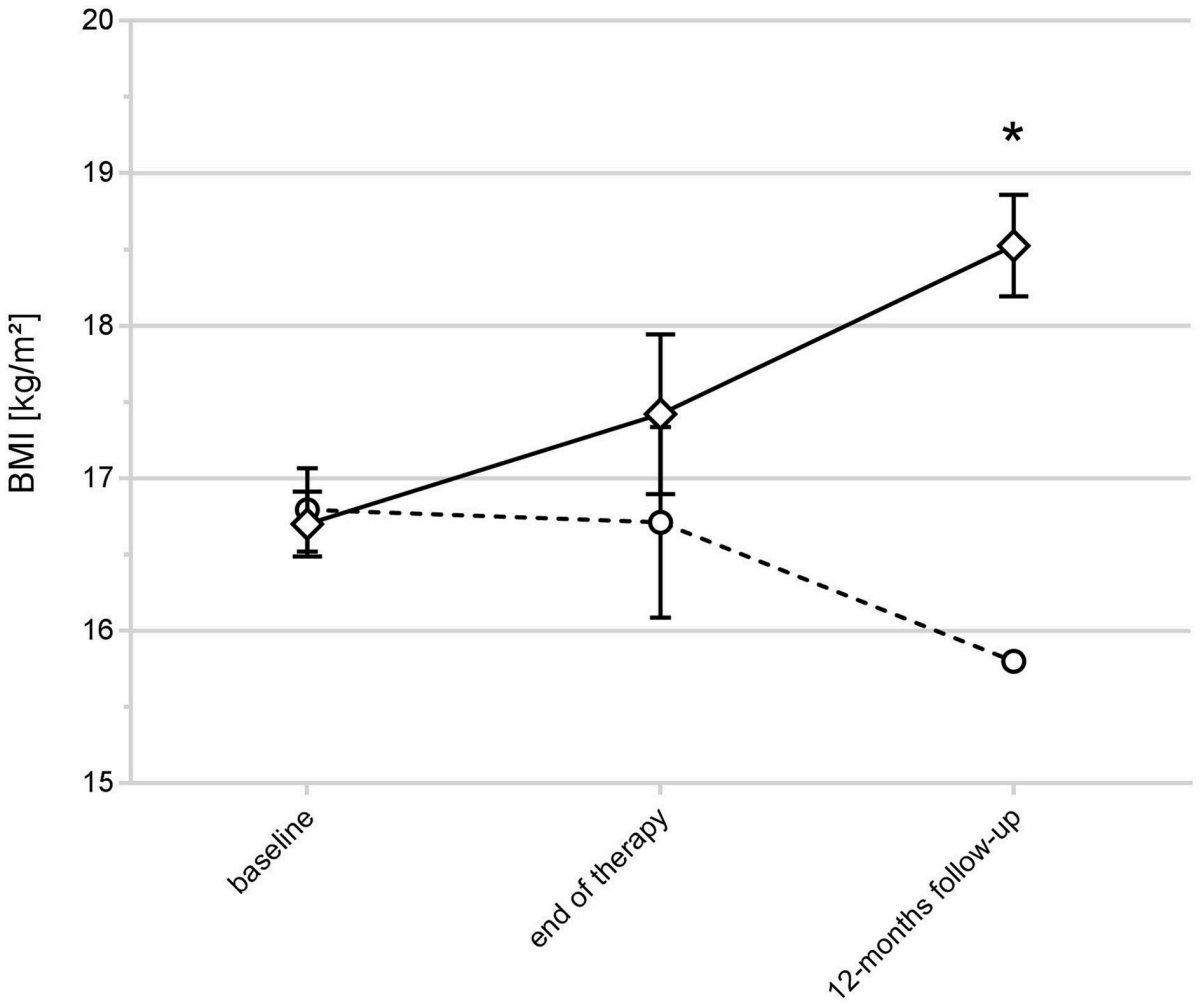
BMI trajectories and *LEP* DNA methylation status in AN with unclassified outcome. The figure shows the BMI trajectories of the replication sample (33 non classified AN patients). Patients with *LEP* DNA methylation levels below the calculated cut-off A showed an increase in BMI over time (indicated by rectangles), those above cut-off A (indicated by circles) had a decrease of the BMI (*ANOVA at the 12-months follow-up: *P* = 0.0142). Raw values are shown; error bars show the standard deviation.

Repeated measurements analysis of variance revealed a significant difference in the BMI trajectories (as an indicator of response/non-response) of the 33 study participants of the replication sample that were not included in the previous analyses: Patients with *LEP* DNA methylation levels below cut-off A showed an increase in BMI over time (response), while those above cut-off A had a decrease of the BMI (non-response). Group differences were significant at the 12-months follow-up (ANOVA: *P* = 0.0142, Figure 4; Supplemental Table 6).

## Discussion

To our knowledge this is the first study reporting changes in DNA methylation of the *LEP* and *LEPR* gene in patients with AN longitudinally during therapy and follow-up. Contrary to our hypothesis, we found a lower DNA methylation of the *LEP* gene promoter in acute AN compared to controls and, in line with our expectations, a DNA hypomethylation of the *LEPR* gene promoter. Both genes exhibited an increase in methylation during therapy, but only the changes in *LEP* gene DNA methylation were marked. We consider the *LEPR* methylation differences as being too small with a too high standard deviation to be biologically significant.

The key finding of our study was that patients with full recovery after therapy showed a significantly more pronounced low baseline DNA methylation of the *LEP* gene with an increase of more than 8% at the 12-months follow-up, while patients with a partial syndrome AN after therapy started with a higher methylation which increased less. The DNA methylation of non-responders with a full syndrome AN after therapy remained stable over time. Finally, we tested the predictive properties of lower *LEP* DNA methylation and were able to distinguish BMI trajectories in a previously unclassified group of patients via an ROC-curves derived cut-off from the analysis of the full recovery AN vs. the full syndrome AN group.

To date, only one pilot study has assessed *LEP* methylation in AN, reporting no differences between patients and controls (13). This pilot study compared 45 patients with current or past AN with age-matched healthy controls. The authors themselves stated, that at the time of measurement it was not clear which participants were currently ill or recovered. Therefore, the lack of difference in their study may partially be explained by the normalization of *LEP* DNA methylation in patients with past AN. In addition, our study included three times more patients and finally, we employed state-of-the-art statistical modeling of promoter DNA methylation compared to simple comparisons of the promoter mean DNA methylation.

Cross-sectional studies on epigenetics in AN like the above mentioned, have not been able to distinguish between alterations due to starvation that would be similar in non-AN fasting states and AN-specific alterations that remain after weight restoration.

Our present findings regarding *LEP* DNA methylation are unlikely to be solely related to BMI or nutritional status, as lower *LEP* DNA methylation was present in patients with later full recovery, who had a higher baseline BMI, compared to partial/full syndrome AN patients who had a lower baseline BMI. Furthermore, there was no correlation between BMI and *LEP* DNA methylation. Additionally, *LEPR* methylation was nearly unchanged during therapy with only a marginal increase over time, thus serving as a “control gene” in our cohort.

Nevertheless, at a first glance the lower DNA methylation of the *LEP* gene in AN is counterintuitive, as one expects a lower DNA methylation to be associated with a higher leptin expression: First, the proximal promoter DNA demethylation of the *LEP* gene has been shown to induce leptin expression in mature adipocytes (39, 40). Second, *LEP* DNA methylation seems to correlate between adipocytes and blood cells (41) and third, circulating leptin levels have been reported to be inversely correlated with blood *LEP* DNA methylation (42, 43). Of note, the proximal promoter region of the *LEP* gene covers another Cpg Island which was not part of our current investigation, while the here studied fragment has been previously shown to have a positive correlation with leptin expression in patients with ethyltoxic cirrhosis (44). In line with the usually inverse relationship between DNA methylation and gene expression, lower *LEP* DNA methylation has been mainly reported in obese patients (31). One study reported lower baseline *LEP* DNA methylation in obese patients who responded to a low-calory diet (45), while another one found no differences in white adipose tissue *LEP* methylation before and after bariatric surgery-induced weight loss (46). Of note, in one of our own studies, we found a higher DNA methylation of the *LEP* promoter in pre-bariatric surgery patients (accompanied by higher leptin serum levels) compared to post-bariatric surgery patients (Wilhelm et al. in preparation).

One could speculate that the unexpected direction of *LEP* DNA methylation is related to these extreme nutritional conditions and could present a counter-regulation of the epigenetic system via a regulating feedback mechanism (e.g., hyperleptinemia inducing higher *LEP* methylation to downregulate leptin expression).

In this regard, it is interesting that in our study patients with the lowest *LEP* DNA methylation were the ones fully responding to therapy. One explanation could be that weight gain was easier to achieve for these patients. In line with this hypothesis are the findings from Kuroda and colleagues (47), who reported the downregulation of *LEP* DNA methylation to be the crucial step for leptin expression mediated adipogenesis in 3T3-L1 cells.

One could speculate that the dynamic change of *LEP* DNA methylation in patients with full recovery could indicate, that a higher adaptability of *LEP* DNA methylation- and expression- is favorable for a better therapy outcome in AN.

Though recent studies reported no value of leptin expression itself as a marker for therapy outcome (48), a study by Baskaran and colleagues (49) showed that the expression of leptin in AN is much more complex than usually considered. They demonstrated that the hypoleptinemia in patients with AN originates from lower amplitudes and mass of the pulsatile leptin expression, rather than the basal one. This lower pulsatile leptin secretion was associated with a higher severity of disordered eating thoughts and behavior. Accordingly, future studies should explore the impact of pulsatile leptin secretion and it's relation to the *LEP* DNA methylation status in blood.

Another possible explanation for our finding could be that low *LEP* DNA methylation points to a subgroup of patients especially sensitive to psychotherapeutic treatment.

Interestingly, *LEP* DNA methylation status in infants has been shown to be influenced by pre- and perinatal factors, like maternal obesity or infant growth. For example, a higher birth weight was associated with a lower *LEP* DNA methylation in early childhood (42), while prenatal exposure to the Dutch famine was associated to a higher *LEP* DNA methylation in the male offspring (50).

As adverse pre- and perinatal factors, possibly reflecting brain damage, predispose for AN, epigenetics and *LEP* DNA methylation could serve as a molecular memory of previous gene-environment interactions (51, 52). These epigenetic and *LEP* alterations in AN could also be important during adolescence, the typical age of onset of AN and a critical environmental period with a major impact on brain maturation and adult behavior (53). In this context it is important to note, that leptin seems to play a role in brain development, cognitive functioning and emotional processes (54).

Our study has several strengths, for example the defined treatments and objective outcomes via weight measurements in a controlled clinical trial. Additionally, our normal-weight controls were age and height matched and sampled at two different timepoints to ensure DNA methylation stability. However, our study is limited in our ability to understand the mechanisms behind the observed associations. For example, we only measured DNA methylation in whole blood and some of the observed changes could have been influenced by cell composition. We also acknowledge that, despite the described association of *LEP* DNA methylation levels in different cell types, we cannot completely translate our findings to tissue-specific DNA methylation and did not measure leptin expression

For these questions it would be useful to measure total body fat, *LEP* DNA methylation in adipocytes, as well as the expression of leptin and other factors of the appetite-regulating system. Further work will also be required to elucidate if the observed alterations point to a subgroup of AN patients with a special psychopathological state, exposure to early-life or adolescent adversity, nutritional specifications or a combination of these factors. Lastly, our results are limited by the shortcomings of percent methylation determination by Sanger-Sequencing of PCR products e.g., due to PCR bias.

In summary, we report for the first time an association between lower DNA methylation of the *LEP* gene in patients with AN and response to psychotherapy.

Further knowledge about the impact of epigenetics and particularly *LEP* DNA methylation on the pathophysiology and course of AN could help to identify vulnerable individuals or AN subgroups and thus lead to more targeted treatment options.

## Ethics Statement

The study adhered to the Declaration of Helsinki (1964) and its later amendments. Independent research ethics committees at every participating center approved the ANTOP study. Approval for the control study was obtained at the Ethics Committee of the University of Hannover (Permit Number 6,427). Written informed consent was obtained from all patients and controls after the procedures had been fully explained to them and prior to their inclusion in the study.

## Author Contributions

VB, AB, MdZ, WH, KG, SH, AD, MB, AZ, SZ, and HF were involved in the conception and design of the study. All authors were involved in data acquisition. Laboratory analyses were performed by VB, AB, and KJ. AN and HF performed the statistical analysis. AN, HF, and SZ were involved in data analysis and interpretation. AN and HF drafted the article. All authors were involved in revising it critically for important intellectual content. All authors approved the final version of the manuscript.

### Conflict of Interest Statement

The authors declare that the research was conducted in the absence of any commercial or financial relationships that could be construed as a potential conflict of interest. The handling editor declared a shared affiliation, though no other collaboration, with several of the authors SZ and KG at the time of the review.
