# Supplementary material for: Association of Leptin Gene DNA Methylation With Diagnosis and Treatment Outcome of Anorexia Nervosa
Source: Front Psychiatry. 2019 Apr 11;10:197. doi: 10.3389/fpsyt.2019.00197 (PMC6470249; doi:10.3389/fpsyt.2019.00197)

Supplementary Material

Association of leptin gene DNA methylation with diagnosis and treatment outcome of anorexia nervosa

**Alexandra Neyazi, Vanessa Buchholz, Alexandra Burkert, Thomas Hillemacher, Martina de Zwaan, Wolfgang Herzog, Kirsten Jahn, Katrin Giel, Stephan Herpertz, Christian A. Buchholz, Andreas Dinkel, Markus Burgmer, Almut Zeeck, Stefan Bleich, Stephan Zipfel, Helge Frieling***

*** Correspondence:** Prof. Dr. med. Helge Frieling; [Frieling.helge@mh-hannover.de](mailto:Frieling.helge@mh-hannover.de)

**Supplemental Table S1A.** Primer sets and cycler conditions for nested PCR of the *LEP* promoter on Chromosome 7: 128240654 to 128241479*

|  | Primer | Primer sequence | PCR thermal profile | No. of cycles |
| --- | --- | --- | --- | --- |
| PCR I | LEP_F1  LEP_R1 | TTT GGG GTG TTA GTT AGA GAT  GAT AAC CTT CTA TCT AAC TAA AAC | 95°C - 15min  97°C - 1min  95°C - 30sec  72°C - 45sec  68°C - 1min  95°C - 30sec  57°C - 45sec  65°C - 1.5min  65°C - 5min  12°C - ∞ | 15x      25x |
| PCR II | LEP_F2  LEP_R2 | TTAGAGAAGGGGTGGGATTTTAG  TACATCCCTCCTAACTCAATTTCC | „ | „ |
| seqPCR | LEP_R2 | TACATCCCTCCTAACTCAATTTCC | 96°C 1min  96°C 10sec  50°C 5sec  60°C 4min  12°C ∞ | 28x |

* According to GRCh38.p10 Primary Assembly (Genome Reference Consortium Human Build 38

patch release 10; Date: 2017/01/06) GenBank assembly accession: GCA_000001405.25 (latest)

**Supplemental Table S1b.** Primer sets and cycler conditions for nested PCR of the *LEPR* promoter and exon I on Chromosome 1: 65420297 to 65420951*

|  | Primer | Primer sequence | PCR thermal profile | No. of cycles |
| --- | --- | --- | --- | --- |
| PCR I | LEPR_F1  LEPR_R1 | GGATTAGTAGGGGAGGTTTT  CTCCACCCACACCCCAAA | 95°C - 15min  95°C - 30sec  65°C - 1.5min  72°C - 2.5min  95°C - 30sec  65°C - 45sec  72°C - 1min  72°C - 4min  12°C - ∞ | 5x      28x |
| PCR II | LEPR_F2  LEPR_R2 | GGAGGAGTTTTGTATTGTTTG  AAAATAACAACCCCACCACA | „ | „ |
| seqPCR | LEPR_R2 |  | 96°C 1min  96°C 10sec  50°C 5sec  60°C 4min  12°C ∞ | 28x |

* According to GRCh38.p7 Primary Assembly (Genome Reference Consortium Human Build 38

patch release 7; Date: 2016/03/21) GenBank assembly accession: GCF_000001405.33 (latest)

# Supplemental Statistical Tables S2. **Testing best model fit for different covariance structures for CpGs as repeated measures**

**(A) Baseline (patients and controls)**

1. ***LEP* Empty model; CpG within subject as repeated measure**

***SPSS-Syntax: MIXED meth***

***/CRITERIA=CIN(95) MXITER(100) MXSTEP(10) SCORING(1) SINGULAR(0.000000000001) HCONVERGE(0,***

***ABSOLUTE) LCONVERGE(0, ABSOLUTE) PCONVERGE(0.000001, ABSOLUTE)***

***/FIXED=| SSTYPE(3)***

***/METHOD=REML***

***/PRINT=SOLUTION***

***/REPEATED=CpG | SUBJECT(subject) COVTYPE(xy)***

| **Covariance structure(xy)** | **AIC** | **No of parameters** |
| --- | --- | --- |
| **AD1** | **6074.30** | **64** |
| **AR1** | **3506.18** | **3** |
| **ARMA11** | **3782.76** | **4** |
| **CS** | **3237.92** | **3** |
| **DIAG** | **3388.10** | **42** |
| **FA1** | **2923.56*** | **43** |
| **HF** | **1668.90*** | **43** |
| **ID** | **2774.55** | **2** |
| **TP** | **4800.14** | **42** |
| **UN** | **8403.48** | **529** |

**AIC. Akaike’s information criterion. AD1**. First-order ante-dependence. **AR1**. First-order autoregressive. **ARMA11**. Autoregressive moving average (1,1). **CS**. Compound symmetry. **DIAG**. Diagonal. **FA1**. First-order factor analytic with constant diagonal offset (d≥0).; **HF**. Huynh-Feldt. **ID**. Identity. **TP**. Toeplitz. **UN**. Unstructured. *****no convergence reached

**Scaled identity (ID) was chosen for the following analyses, as it was the model with the lowest AIC and the least parameters reaching convergence within 100 iterations. We tested, if increasing the number of iterations up to 500 lead to a converging model for the HF covariance structure, which did not succeed.**

1. ***LEPR* Empty model; CpG within subject as repeated measure**

***SPSS-Syntax: MIXED meth***

***/CRITERIA=CIN(95) MXITER(100) MXSTEP(10) SCORING(1) SINGULAR(0.000000000001) HCONVERGE(0,***

***ABSOLUTE) LCONVERGE(0, ABSOLUTE) PCONVERGE(0.000001, ABSOLUTE)***

***/FIXED=| SSTYPE(3)***

***/METHOD=REML***

***/PRINT=SOLUTION***

***/REPEATED=CpG | SUBJECT(subject) COVTYPE(xy)***

| **Covariance structure(xy)** | **AIC** | **No of parameters** |
| --- | --- | --- |
| **AD1** | **15 747.18** | **66** |
| **AR1** | **11 922.34** | **3** |
| **ARMA11** | **12 033.79** | **4** |
| **CS** | **11 471.03** | **3** |
| **DIAG** | **15 185.71** | **34** |
| **FA1** | **11 371.10*** | **35** |
| **HF** | **11 588.04*** | **35** |
| **ID** | **11 269.95** | **2** |
| **TP** | **12 255.90** | **34** |
| **UN** | **16 152.58*** | **562** |

***AIC. Akaike’s information criterion. AD1****.* First-order ante-dependence. ***AR1****.* First-order autoregressive. ***ARMA11****.* Autoregressive moving average (1,1). ***CS****.* Compound symmetry. ***DIAG****.* Diagonal. ***FA1****.* First-order factor analytic with constant diagonal offset *(*d*≥0*)*.;* ***HF****.* Huynh-Feldt. ***ID****.* Identity. ***TP****.* Toeplitz. ***UN****.* Unstructured. *****no convergence reached

**Scaled identity (ID) was chosen for the following analyses, as it was the model with the lowest AIC and the least parameters reaching convergence.**

**(B) Three timepoints (patients only)**

1. ***LEP* Empty model; timepoint*CpG within subject as repeated measure**

***SPSS-Syntax: MIXED methylation By timepoint***

***/CRITERIA=CIN(95) MXITER(100) MXSTEP(10) SCORING(1) SINGULAR(0.000000000001) HCONVERGE(0,***

***ABSOLUTE) LCONVERGE(0, ABSOLUTE) PCONVERGE(0.000001, ABSOLUTE)***

***/FIXED=| SSTYPE(3)***

***/METHOD=REML***

***/REPEATED=timepoint*CpG | SUBJECT(subject) COVTYPE(xy).***

| **Covariance structure(xy)** | **AIC** | **No of parameters** |
| --- | --- | --- |
| **AD1** | **3262.52** | **194** |
| **AR1** | **1297.96** | **5** |
| **ARMA11** | **1541.84** | **6** |
| **CS** | **861.14** | **5** |
| **DIAG** | **1434.09** | **99** |
| **FA1** | **342,16*** | **100** |
| **HF** | **666.80*** | **100** |
| **ID** | **528.28** | **4** |
| **TP** | **2457.96** | **99** |
| **UN** | **#** |  |

**AIC. Akaike’s information criterion. AD1**. First-order ante-dependence. ***AR1****.* First-order autoregressive. ***ARMA11****.* Autoregressive moving average (1,1). ***CS****.* Compound symmetry. ***DIAG****.* Diagonal. ***FA1****.* First-order factor analytic with constant diagonal offset *(*d*≥0*)*.;* ***HF****.* Huynh-Feldt. ***ID****.* Identity. ***TP****.* Toeplitz. ***UN****.* Unstructured. *****no convergence reached. #too many parameters to estimate

**Scaled identity (ID) was chosen for the following analyses, as it was the model with the lowest AIC and the least parameters reaching convergence.**

1. ***LEPR* Empty model; timepoint*CpG within subject as repeated measure**

***SPSS-Syntax: MIXED methylation By timepoint***

***/CRITERIA=CIN(95) MXITER(100) MXSTEP(10) SCORING(1) SINGULAR(0.000000000001) HCONVERGE(0,***

***ABSOLUTE) LCONVERGE(0, ABSOLUTE) PCONVERGE(0.000001, ABSOLUTE)***

***/FIXED=| SSTYPE(3)***

***/METHOD=REML***

***/REPEATED=timepoint*CpG | SUBJECT(subject) COVTYPE(xy).***

| **Covariance structure(xy)** | **AIC** | **No of parameters** |
| --- | --- | --- |
| **AD1** | **12 189.35** | **133** |
| **AR1** | **9 361.58** | **4** |
| **ARMA11** | **9 410.38** | **5** |
| **CS** | **9 186.75** | **4** |
| **DIAG** | **11 938.99** | **68** |
| **FA1** | **8 999.55*** | **69** |
| **HF** | **9 015.55*** | **69** |
| **ID** | **9 147.55** | **3** |
| **TP** | **9 516.93** | **68** |
| **UN** | **#** |  |

**AIC. Akaike’s information criterion. AD1**. First-order ante-dependence. **AR1**. First-order autoregressive. **ARMA11**. Autoregressive moving average (1,1). ***CS****.* Compound symmetry. **DIAG**. Diagonal. **FA1**. First-order factor analytic with constant diagonal offset *(*d*≥0*)*.;* **HF**. Huynh-Feldt. **ID**. Identity. **TP**. Toeplitz. **UN**. Unstructured. *****no convergence reached. #too many parameters to estimate

**Supplemental Table S3. Comparison of baseline characteristics of patients remaining in the study until 12-month follow up and patients dropping out of the study/without PSR ratings at 12 month follow-up**

|  | **12-month follow-up completer (n=93)** | **Drop-out/no PSR rating at follow-up (n=36)** | **T/Χ²** | **df** | **P-value** |
| --- | --- | --- | --- | --- | --- |
| Mean *LEP*  methylation | 0.317 ± 0.09 | 0.295 ± 0.07 | 1.258 | 127 | 0.21 |
| Mean *LEPR*  methylation | 0.042 ± 0.024 | 0.044 ± 0.024 | -0.378 | 127 | 0.71 |
| Age, years | 27.23 ± 7.5 | 27.50 ± 7.9 | -0.183 | 127 | 0.86 |
| BMI, T0 | 16.86 ± 0.92 | 16.64 ± 0.98 | 1.193 | 127 | 0.235 |
| Subtype, n (%) |  |  | 0.52 | 1 | 0.82 |
| Binge-purge AN | 46 (49.5) | 17 (47.2) |  |  |  |
| Restrictive AN | 47 (50.5) | 19 (52.8) |  |  |  |

**Supplemental Table S4.** Details of the mixed linear model (II) assessing the influence of timepoint and outcome on *LEP* DNA methylation

| **Predictor** | **F-value (fixed effect)** | **P-value** | **Categories** | **Parameter estimate** | **Standard error** | **T-value** | **df** | **P-value** |
| --- | --- | --- | --- | --- | --- | --- | --- | --- |
| Intercept | 61.70 | <0.001 |  | 43.54 | 6.18 | 7.05 | 5985 | <0.001 |
| timepoint | 8.79 | <0.001 | Baseline | -8.71 | 2.16 | -4.04 | 5985 | <0.001 |
|  |  |  | End of therapy | -2.76 | 1.95 | -1.42 | 5985 | 0.16 |
|  |  |  | 12month FU* | 0 |  |  |  |  |
| Outcome | 0.69 | 0.50 | full syndrome AN | -3.17 | 2.58 | -1.23 | 5958 | 0.22 |
|  |  |  | partial syndrome | -1.68 | 1.94 | -0.86 | 5985 | 0.39 |
|  |  |  | full recovery* | 0 |  |  |  |  |
| Timepoint*outcome | 3.29 | 0.011 | baseline*full syndrome AN | 9.10 | 2.83 | 3.21 | 5985 | 0.001 |
|  |  |  | baseline*partial syndrome | 6.16 | 2.29 | 2.70 | 5985 | 0.007 |
|  |  |  | end of therapy * full syndrome AN | 3.92 | 2.66 | 1.48 | 5985 | 0.14 |
|  |  |  | End of therapy * partial syndrome | 1.96 | 2.21 | 0.89 | 5985 | 0.37 |
| age | 0.11 | 0.75 |  | 0.01 | 0.04 | 0.32 | 5985 | 0.75 |
| BMI | 2.25 | 0.13 |  | -0.43 | 0.28 | -1.50 | 5985 | 0.13 |

*LEP* DNA methylation was the dependent variable. Categories marked with an asterisk (*) and interactions of categories not listed are redundant and parameter estimates were set to 0.

AIC: 23.96; No. of parameters: 12; No. of observations: 93; Estimated covariance parameter (repeated measurements): 5.75 (s.e.:1.1)

**Supplemental Table S5.** Details of the mixed linear model (II) assessing the influence of timepoint and outcome on *LEPR* DNA methylation

| **Predictor** | **F-value (fixed effect)** | **P-value** | **Categories** | **Parameter estimate** | **Standard error** | **T-value** | **df** | **P-value** |
| --- | --- | --- | --- | --- | --- | --- | --- | --- |
| Intercept | 11.52 | <0.001 |  | 7.25 | 2.52 | 2.87 | 6407 | 0.004 |
| timepoint | 4.38 | 0.013 | Baseline | -0.34 | 0.87 | -0.39 | 6407 | 0.70 |
|  |  |  | End of therapy | 0.35 | 0.79 | 0.44 | 6407 | 0.66 |
|  |  |  | 12month FU* | 0 |  |  | 6407 |  |
| Outcome | 0.064 | 0.94 | full syndrome AN | 0.29 | 1.01 | 0.28 | 6407 | 0.78 |
|  |  |  | partial syndrome | -0.24 | 0.77 | -0.31 | 6407 | 0.76 |
|  |  |  | full recovery* | 0 |  |  | 6407 |  |
| Timepoint*outcome | 2.28 | 0.059 | baseline*full syndrome AN | -1.28 | 1.14 | -1.13 | 6407 | 0.26 |
|  |  |  | baseline*partial syndrome | 0.73 | 0.92 | 0.79 | 6407 | 0.43 |
|  |  |  | end of therapy * full syndrome AN | 0.19 | 1.06 | 0.18 | 6407 | 0.86 |
|  |  |  | End of therapy * partial syndrome | 0.14 | 0.90 | 0.16 | 6407 | 0.88 |
| age | 0.012 | 0.91 |  | 0.002 | 0.018 | 0.11 | 6407 | 0.91 |
| BMI | 1.01 | 0. 32 |  | -0.12 | 0.12 | -1.01 | 6407 | 0.32 |

*LEPR* DNA methylation was the dependent variable. Categories marked with an asterisk(*) and interactions of categories not listed are redundant and parameter estimates were set to 0.

AIC: 23.96; No. of parameters: 12; No. of observations: 93; Estimated covariance parameter (repeated measurements): 5.75 (s.e.:1.1)

**Supplemental Table S6**. ANOVA table of PSR unclassified patients: Effect of *LEP* DNA methylation (cut-off A) on BMI trajectories

| **source** | **SS** | **DF** | **F (DFn, DFd)** | **P-value** |
| --- | --- | --- | --- | --- |
| Interaction | 11.66 | 2 | F (2.52) = 3.647 | P = 0.0330 |
| timepoint | 1.048 | 2 | F (2.52) = 0.3279 | P = 0.7219 |
| Cut-off A* | 10.29 | 1 | F (1.52) = 6.436 | P = 0.0142 |
| Residual | 83.10 | 52 |  |  |

BMI was the dependent variable. ANOVA at the 12-months follow-up, *< 31.25% *LEP* DNA methylation rate vs. ≥ 31.25% *LEP* DNA methylation rate.

**Supplemental Figure S1a.** *LEP* gene structure and position of the promoter fragment

***LEP* F1 Primer**

1 AGCCCTCCAGAGAGCGTGCACTC**CCTGGGGTGCCAGCCAGAGAC**AACTTGCCCTGAGGCTTG

***LEP* F2 Primer**

63 GAACTCGATTCTCCGCGTG**CCAGAGAAGGGGTGGGACTTCAG**AACCCCCAACCCCGCAATCT

125 GGGTCGGGGAGCCTGGCGCACTGCGGGCCGCTCCCTCTAACCCTGGGCTTCCCTGGCGTCCA

187 GGGCCGTCGGGGCCGAGTCCCGATTCGCTCCCACCCCGAAGCCGCGCCAGGACCAACGAGGG

249 CGCAGCCGTATGCCCCAGCCCGCTCCGCGGAGCCCCTCACAGCCACCCCCGCCCCGACCGCG

311 CCCCGCGCGGCTCGAAGCACCTTCCCAAGGGGCTGGTCCTTGCGCCATAGTCGCGCCGGAGC

373 CTCTGGAGGGACATCAAGGATTTCTCGCTCCTACCAGCCACCCCCAAATTTTTGGGAGGTAC

435 CCAAGGGTGCGCGCGTGGCTCCTGGCGCGCCGAGGCCCTCCCTCGAGGCCCCGCGAGGTGCA

497 CACTGCGGGCCCAGGGCTAGCAGCCGCCCGGCACGTCGCTACCCTGAGGGGCGGGGCGGGAG

559 CTGGCGCTAGAAATGCGCCGGGGCCTGCGGGGCAGTTGCGCAAGTTGTGATCGGGCCGCTAT

***Exon 1***

621 AAGAGGGGCGGGCAGGCATGGAGCCCCGTAGGA**ATCGCAGC**GCCAGCGGTTGCAAGGTAAGG

683 CCCCGGCGCGCTCCTTCCTCCTTCTCTGCTGGTCTTTCTTGGCAGGCCACAGGGCCCCACAC

***LEP* R2 Primer**

745 AACTCTGGATCCCGG**GGAAACTGAGTCAGGAGGGATGCA**GGGCGGATGGCTTAGTTCTGGAC

***LEP* R1 Primer**

807 TATGATAGCTTTGTACCGA**GTTCTAGCCAGATAGAAGGTTACC**GGGAGCTGGGGAGCGTTGG

**Fig. S1a** shows the investigated promoter region of the *LEP* gene. The fragment covered 702 bp and 41 CpG´s (highlighted in grey). Primers are underlined, the transcription start site (TSS) is marked with an arrow.

**Supplemental Figure S1b.** *LEPR* gene structure and position of the exon/promoter fragment

***LEPR* F1 Primer *LEPR* F2 Primer**

1 CG**GGACCAGCAGGGGAGGCCTC**CGCGAGCTAGG**GGAGGAGCTCTGCATTGTCTG**GGGCGGGG

63 CTTCGAGTAGCGGTAGCGAGGTCTGGAGCCGCAGGCGCTGCCTCCGCGAGGTAGGGGAGGAG

125 CTCTGTACTGTCAGGGGCGGGGCTCTGAGTAGCGAGGCCGGGTCTGAACTATCAGGCGCGGC

187 CTCTGCGAGCTGGGGGCGGGGTTCTGCACCGGCGGGGGCGGGGTTGTGAGTGGTGGGGGCGG

***TSS***

249 GGTCTGGAGCAGCAGGCGCCGCGTTTGCGAGCTAAGGTCGGA***GTTCTGCAC*CGGCGGGGGCG**

311 **GGGCTCTGCGTGGCCGGGGCGGGCTCGGGATCCGCGGGGCGACTCCCGGTCTGGCTTGGGCA**

373 **GGCTGCCCGGGCCGTGGCAGGAAGCCGGAAGCAGCCGCGGCCCCAGTTCGGGAGACATGGCG**

***LEPR* R2 Primer**

435 **GGCGTTAAAG**GTACATCGCGGTCCCCGGCTCGCTTGTCG**TGTGGTGGGGTTGCCACCTC**CGT

497 TCCGGTCAAGCCTGGGGCTGCGCCTTCCGCGCGCCGTTGGGGAACGGCCTCACCACCCTTCC

***LEPR* R1 Primer**

559 CGCCTCTCCGGTTCGGGAGGCGATCGACCGCTCCCTTCGTCC**CTTGGGGTGTGGGTGGAG**CG

**Fig. S1b** shows the investigated promoter and exon region of the *LEPR* gene. The fragment covered 460 bp and 45 CpG´s (highlighted in grey). Primers are underlined, the transcription start site (TSS) is marked with an arrow, the exon in bold font.

**Supplemental Figure 2A/B.** ROC curve and cumulative distribution diagram: Leptin gene DNA methylation levels and differentiation of response type

**Fig. S2A.**


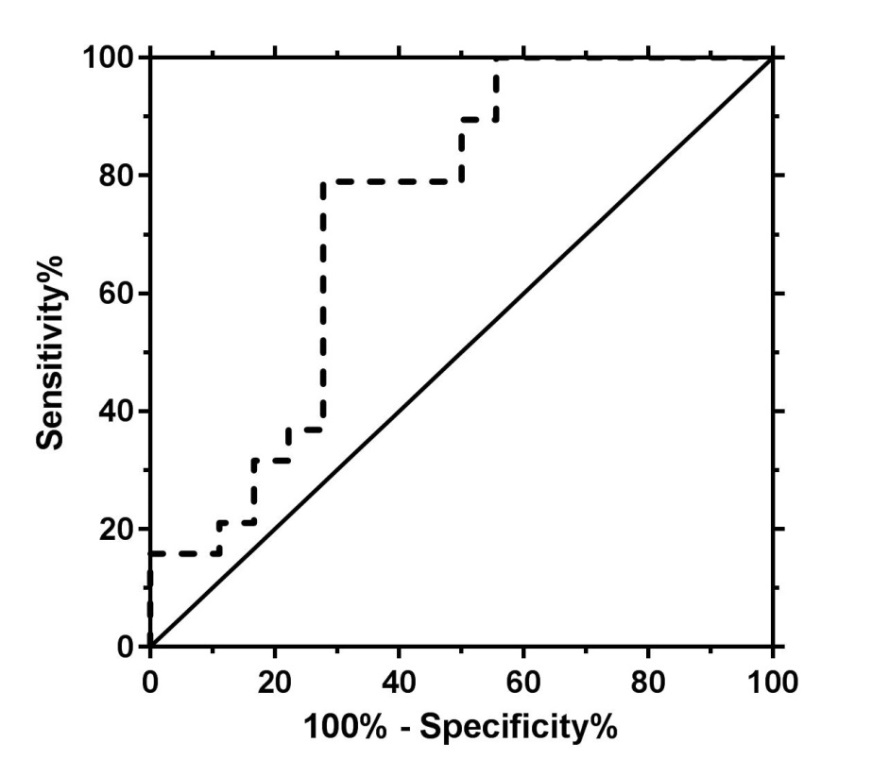


**Fig. S2A** illustrates the Receiver operating characteristic (ROC) curve analysis to evaluate the efficiency of *LEP* DNA methylation levels in differentiating full recovery AN patients from patients full syndrome AN with an area under the curve (AUC) of 0.737 (s.e.: 0.086, P=0.014).

**Fig. S2b.**

**
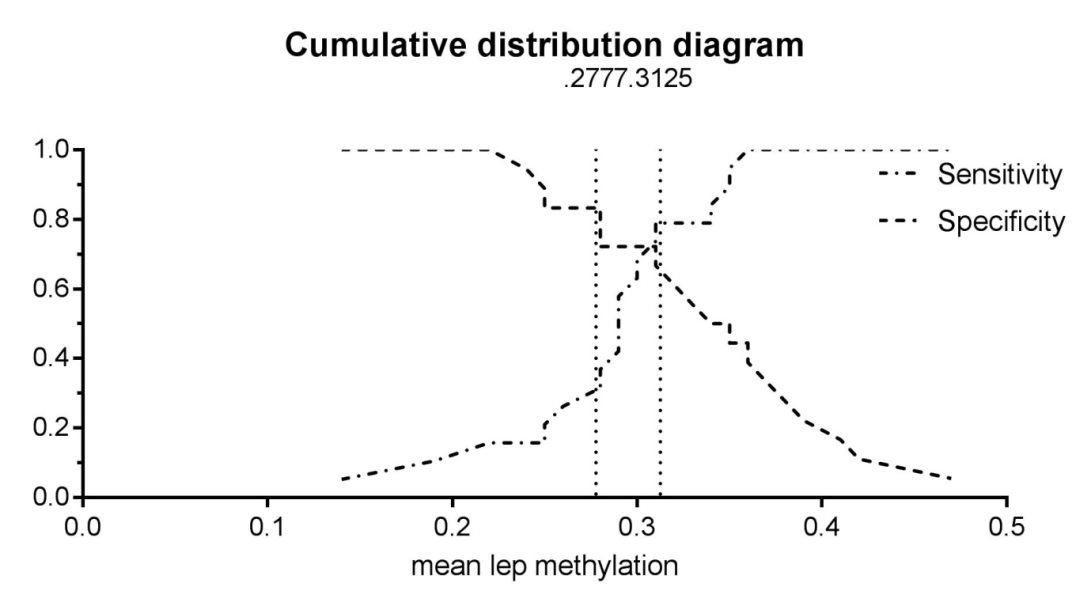
**

**Fig. S2b** shows the cumulative distribution diagram of two cut-off values with a maximized AUC (Youden’s Index). Cut-off A showed the best combined sensitivity and specificity at 0.3125(31.25%) mean *LEP* DNA methylation and cut-off B (0.2777(27.77%) mean *LEP* DNA methylation) had the highest sensitivity at specifity of 0.8.

**Supplemental Figure 3.**


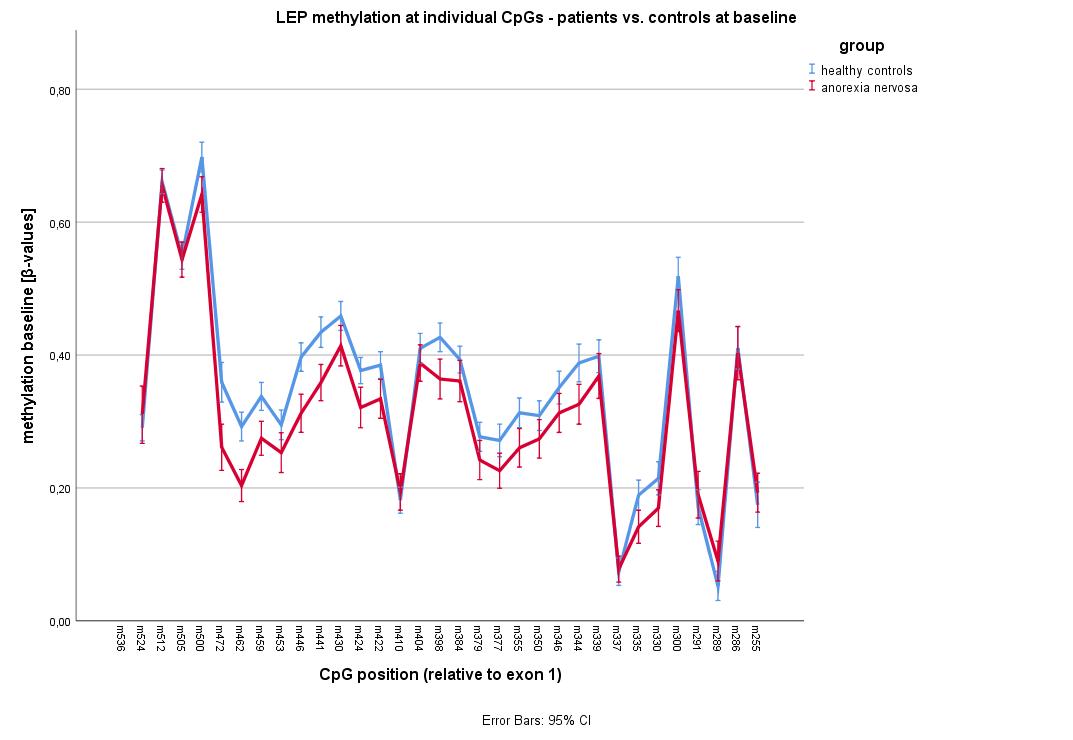


**Supplemental Figure 4A.**


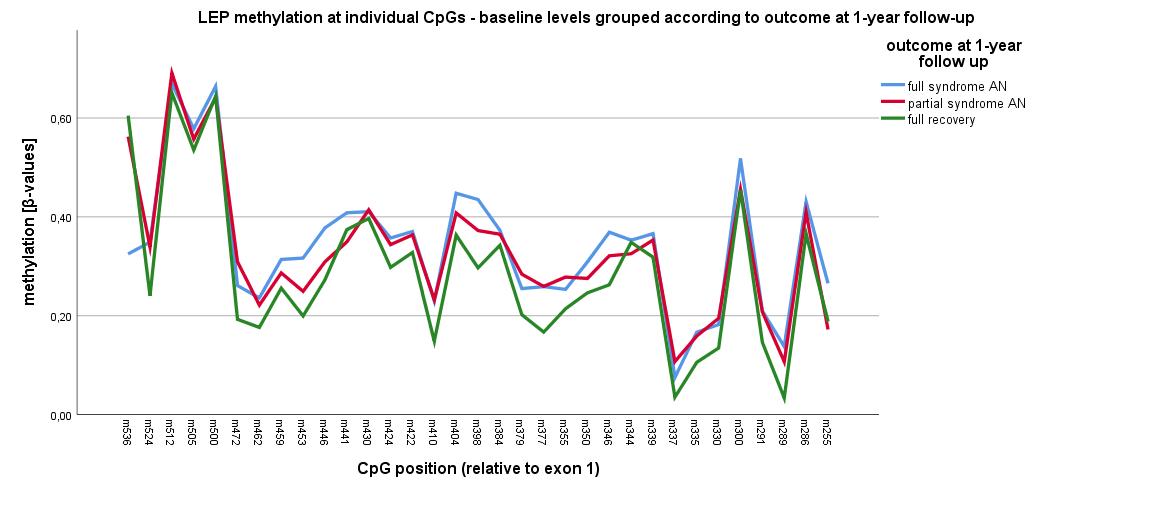


**Supplemental Figure 4B.**

**
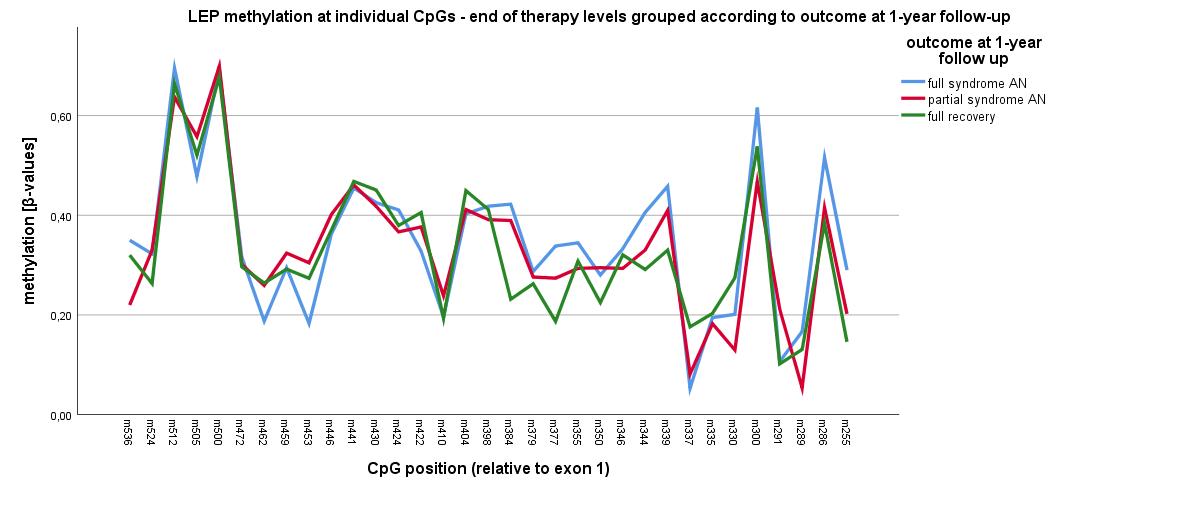
**

**Supplemental Figure 4C.**


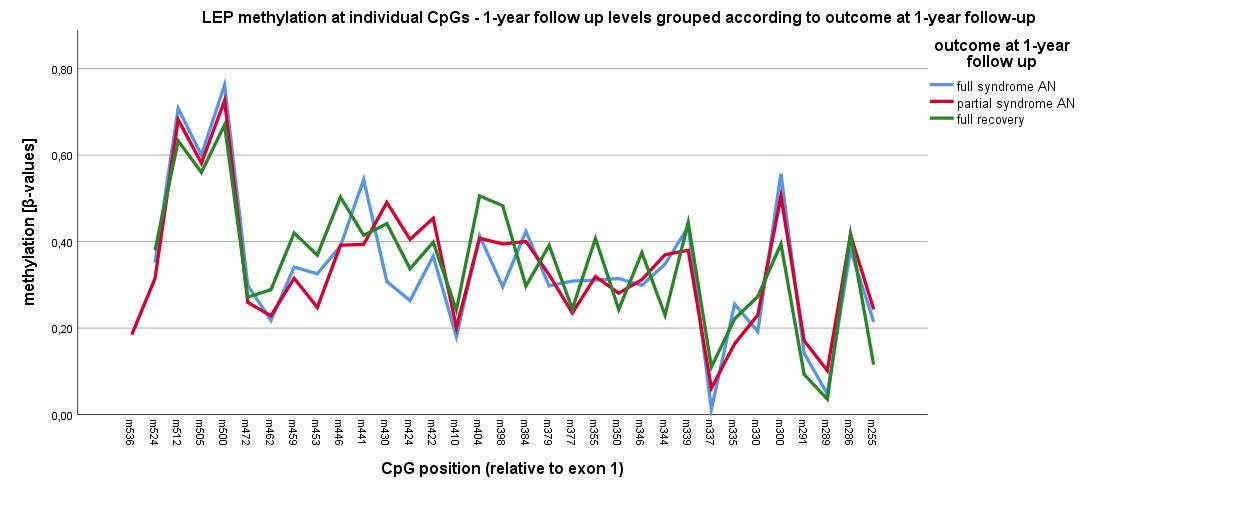

Supplement: Supplementary file 1 [file Data_Sheet_1.docx]
